# Sustaining the HIV/AIDS response: PEPFAR's vision

**DOI:** 10.1002/jia2.26192

**Published:** 2023-11-30

**Authors:** John Nkengasong, Michael Ruffner, Maureen Bartee, Ingrid T. Katz, Michael J. A. Reid

**Affiliations:** ^1^ Bureau of Global Health Security and Diplomacy President's Emergency Plan for AIDS Relief Washington DC USA; ^2^ Department of Medicine Brigham Women's Hospital, Harvard Medical School Boston Massachusetts USA; ^3^ Institute for Global Health Sciences University of California at San Francisco San Francisco California USA

1

Along with partner governments, their civil societies and multilateral organizations, the President's Emergency Plan for AIDS Relief (PEPFAR) has been at the forefront of the global battle against the HIV pandemic for the past two decades. With support from PEPFAR, millions of lives have been saved, including 5.5 million children born HIV‐free [1]. Furthermore, many countries in sub‐Saharan Africa are making significant strides towards achieving the “95‐95‐95 targets” set forth by UNAIDS [2]. The prospect of achieving these targets is within reach, but the challenge is to sustain these achievements over the long term.

This World AIDS Day, we look ahead with optimism to a future where HIV is no longer a public health threat, and every country leads an HIV response that is person‐centred to meet the overall health and wellbeing of its population. Here, we outline three essential priorities for sustaining the HIV/AIDS response, as outlined in PEPFAR's recent five‐year strategy [1]: transformative political leadership, sustained financial commitment and programme innovation. We also highlight critical knowledge gaps that must be addressed to secure these priorities.

From the outset of the global HIV epidemic, it was clear that the HIV/AIDS response required a political commitment to ensure an equitable and whole‐of‐government response. Success stories from countries like Botswana have shown that a sustainable response can be achieved through evidence‐based prevention and treatment strategies supported by non‐discriminatory laws that address stigma and promote equity [3]. Ending the HIV pandemic as a public health threat requires a coordinated and enabling response that centres community voices and removes impediments to populations often facing significant obstacles in access and remaining in care. As such, steadfast, bold and decisive leadership at all levels of government is essential.

To secure the long‐term sustainability of HIV response efforts, it is imperative that all countries fulfil their healthcare funding commitments; sustained domestic investments, which already finance most of the health spending in low‐ and middle‐income countries, are essential regardless of their gross national income [3]. This commitment was underscored in 2001 through the Abuja declaration when African leaders pledged to allocate 15% of their gross domestic product (GDP) to healthcare [4]. A decade later, an HIV investment framework was established to enhance HIV prevention, care and treatment. This framework emphasized the significance of social support systems and broader development goals, such as education and poverty reduction. The United Nations Sustainable Development Goals in 2015 further emphasized this approach, offering a comprehensive strategy to address global challenges while emphasizing the critical role of sustained domestic financial investment in achieving these goals.

As countries reach and sustain the 95‐95‐95 targets, domestic resources must be the cornerstone of HIV financing. Economic growth offers an opportunity for many countries to increase their investment in health [5], but national leaders must demonstrate sufficient political will to allocate these dividends to health services. Increased HIV investments would contribute to broader and sustained economic gains by 2030. A recent UNAIDS report highlighted that South Africa's GDP could rise by 2.8% and Kenya could see its GDP increase by 1.1% by 2030 if domestic HIV targets are met. Nonetheless, countries face many challenges, including mounting national debt burdens [6], competing healthcare priorities, political considerations, and the efficiency of their revenue collection and expenditure systems. Consequently, the specific milestones towards sustainably increasing domestic financing for health will vary from country to country. In the years ahead, global stakeholders, including PEPFAR, UNAIDS and the Global Fund, will need to collaborate closely with partner countries to craft tailored approaches that encourage domestic resource mobilization while considering each country's unique capacity to absorb costs. The collaborative efforts will be geared towards improving both programmatic and financial efficiency, harnessing local capabilities, and aligning government and donor resources effectively. Strategies to expand each country's financial protection programmes will also likely be important as countries advance towards the 95‐95‐95 targets. The example of Vietnam, where modest PEPFAR resources were leveraged to support the Government of Vietnam's expansion of its health insurance scheme to all people living with HIV (PLHIV), illustrates the impact of strategic investments to expand domestic health investments [7].

The rapid scale‐up of the HIV response over the past two decades has been mainly achieved with stand‐alone HIV care and treatment programmes [3]. As countries achieve or exceed the 95‐95‐95 targets, it will be critical to consider how health systems remain responsive to the ongoing healthcare needs of older PLHIV. One critical challenge involves managing hypertension in PLHIV; it is estimated that more than six million PLHIV in Africa have hypertension, and blood pressure control is suboptimal in the vast majority [1]. As such, it is essential that HIV programmes consider how to optimize cardiovascular outcomes for the PLHIV they serve [3]. Similarly, improving mental health outcomes for PLHIV is important to improving HIV outcomes and overall quality of life [8]. As such, PEPFAR has a unique opportunity to innovate with service delivery models to effectively build integrated linkages between HIV service delivery and selected hypertension and mental health delivery. Evaluating the clinical, programmatic and financial impact of these integrated service delivery models can inform how and where partner governments leverage PEPFAR's investment to extend other essential health services, for example cancer screening and diabetes management, while also advancing PEPFAR's commitment to sustainable, country‐run programmes.

PEPFAR has a track record of using science and data to drive programming decisions over the last two decades. By adopting evidence‐based algorithms for HIV testing, prevention and treatment, using standardized laboratory tests and streamlined data monitoring systems, under‐resourced health systems were able to provide care and save more than 25 million lives [9]. To realize the vision of sustained epidemic impact, PEPFAR remains committed to using science and data‐driven solutions, although the scientific challenges are inevitably changing. Moving forward, evidence‐building must focus on determining the best strategies for linking HIV service delivery and selected cardiovascular and mental health services to ensure programmes remain client‐centred. Studies employing a range of analyses including, but not limited to, activity‐based costing [10] and preference elicitation methods [11] are likely to be instrumental in refining both financially sustainable and client‐centred programme strategies and to ensure that both prevention and treatment programmes are accessible and sustainable.

Ultimately, the path to securing and sustaining the UNAIDS 95‐95‐95 targets requires transformative political leadership, sustained financial commitment and programmatic innovation. In all countries, prioritized and sustained efforts are necessary to realize these targets and ensure that no one is left behind. PEPFAR remains committed to supporting partner countries on this journey, working towards a future where each nation leads its HIV response, with PEPFAR's support when necessary.

## AUTHOR INFORMATION

Ambassador Dr. John Nkengasong currently serves as Global AIDS Coordinator and Senior Bureau Official in the Bureau of Global Health Security and Diplomacy.

## AUTHORS’ CONTRIBUTIONS

The first draft was prepared by MJAR. All subsequent drafts were reviewed and revised by all other authors.

## FUNDING

The study was supported by the US Government.

## COMPETING INTERESTS

The authors report no competing interests.
